# Silver-functionalized silica aerogel: towards an understanding of aging on iodine sorption performance

**DOI:** 10.1039/c8ra05137b

**Published:** 2018-09-12

**Authors:** Josef Matyáš, Eugene S. Ilton, Libor Kovařík

**Affiliations:** Pacific Northwest National Laboratory Richland WA 99354 USA Josef.Matyas@pnnl.gov

## Abstract

The silver-functionalized silica aerogel (Ag^0^-aerogel) is being developed for the removal and sequestration of iodine compounds from the off-gas of a nuclear fuel reprocessing plant. This material shows high selectivity and sorption capacity for iodine. However, its sorption performance decreases when exposed to air containing H_2_O and NO_*x*_ at 150 °C for extended periods of time. This phenomenon is referred to as “aging” and simulates exposure of the sorbent during plant idling. This extensive study of unaged and aged samples of Ag^0^-aerogel with and without iodine revealed that decreased efficiency of I capture after NO-aging can be attributed to an increase in size of silver nanoparticles and by the formation of free sulfate on their surfaces from oxidized thiol groups. The smaller reactive surface areas of bigger particles and thin sulfate layer on particle surfaces prevented a complete utilization of the silver. By contrast, formation of silver sulfate appears to be the main factor in decreasing the sorption capacity for samples aged in dry or humid air. It is hypothesized that a short period exposure of the Ag^0^-aerogel to a reducing gas stream would reduce oxidized silver back to metal and sulfate to sulfide. This may recover the sorption performance of Ag^0^-aerogel close to original levels.

## Abstract 2

This manuscript elucidates the mechanism responsible for a decrease of iodine-sorption performance for Ag^0^-functionalized silica aerogel in the reprocessing off-gas streams.

## Graphical abstract

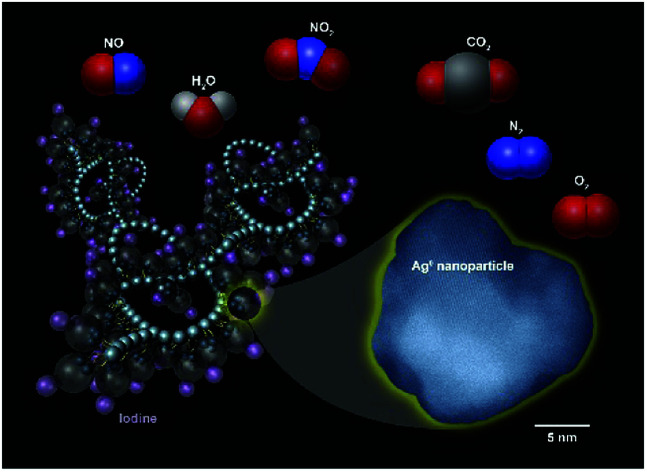

## Introduction

The Fukushima Daiichi accident in 2011 stirred up vigorous discussion on the future of nuclear power. Since then, a number of countries around the world have departed from nuclear power and are transitioning towards renewable energies. However, a large, and still growing, inventory of spent nuclear fuel (SNF) is temporarily stored at nuclear power plants. One solution would be to reprocess this fuel and extract its fission products, such as the uranium-235 and the plutonium-239 for recycle. This process also enables the recovery of chemically similar waste components and their immobilization in the waste forms that will match the waste stream chemistry and target disposal environment. However, reprocessing also results in the release of various gaseous fission products from the fuel into the off-gas system, with radioiodine (^129^I_2(g)_) being of particular concern because of its long half-life (1.6 × 10^7^ years) and the potential for biological processes to concentrate iodine.^1^

A large inventory of solid sorbents such as mordenite (AgZ) or faujasite (AgX),^2^ chalcogen-based aerogels,^3–5^ metal–organic frameworks,^6,7^ granular activated carbon,^8^ graphene powders/aerogels,^9^ copper metal,^10^ bi-compounds,^11^ Ag^0^-functionalized silica aerogels,^12,13^ Ag-impregnated Al_2_O_3_,^14^ and Ag-impregnated SiO_2_ (ref. 15) have been investigated as getters for I_2(g)_, including porous materials functionalized with different metals: Ag, Bi, Cd, Cu, Hg, Mn, Pb, Pd, Sb, Sn, and Tl.^3,4,11,16–22^ However, owing to strong chemisorption for iodine, the leading approach to capturing radioactive iodine from reprocessing off-gas is using silver-containing adsorbents.

Currently, the U.S. does not reprocess SNF. However, to meet licensing requirements, any future reprocessing facilities would require an efficient capture of radioiodine from the off-gas streams. The leading candidate for radioiodine capture in the U.S. is reduced silver mordenite (Ag^0^Z) with the primary alternative option being silver-functionalized silica aerogel (Ag^0^-aerogel).^12^ These materials are required to maintain high sorption capacity and selectivity for iodine when a reprocessing plant is idling and they are exposed to heated air containing water vapor and NO_*x*_. Ideally, sorption performance should not decrease significantly, as that would create a need for more material and either large filters or more frequent filter changes. Extensive studies of these sorbents indicated that the iodine sorption capacity for Ag^0^Z, and somewhat for Ag^0^-aerogel, decreased markedly when exposed to gas streams containing H_2_O and NO_*x*_ at elevated temperature (150 °C) for extended periods of time. This phenomenon is referred to as “aging” of the sorbents. Table 1 summarizes the results from “aging” tests in which the sorbents were aged in different environments for different time and then subsequently loaded with iodine.

**Table 1:** Loss of iodine loading capacity (in relative mass percent) for Ag^0^-aerogel and Ag^0^Z after aging under different gas environments at 150 °C

Aging environment	Iodine capacity for unaged material (mg I g^−1^)		Aging time (months)	Iodine capacity for aged material (mg I g^−1^)	
	Ag^0^-aerogel	Ag^0^Z		Ag^0^-aerogel	Ag^0^Z
Flowing dry air	408	90	6	320 (22)^c^	54 (40)^c^
Flowing humid air	307	97	6	239 (22)^c^	39 (60)^c^
Static dry air 2 vol% NO_2_	331	59	4^a^, 2^b^	283 (15)^c^	41 (30)^c^
Flowing dry air 1 vol% NO	290	97	2	165 (43)^c^	15 (85)^c^

a Ag^0^-aerogel was aged for 4 months.

b Ag^0^Z was aged for 2 months.

c Relative mass percent loss of iodine.

The Ag^0^-aerogel retained high selectivity and sorption capacity for I_2(g)_ even after a long-term exposure to dry/humid air^23,24^ and dry air containing 2 vol% NO_2_.^25^ Aging in dry or humid air over a period of six months resulted in a decrease in iodine sorption capacity of 22 relative mass percent. Aging for four months in dry air containing 2 vol% NO_2_ decreased iodine sorption capacity by 15 relative mass percent. However, exposure of Ag^0^-aerogel to 1 vol% NO at 150 °C for two months decreased sorption capacity by 43 relative mass percent.^26^ Interestingly, in spite of a relatively large loss of sorption capacity, the sorption performance of Ag^0^-aerogel was not as much affected by aging as was the Ag^0^Z. The exact same test with Ag^0^Z resulted in an iodine capacity loss of 85 relative mass percent.^27^

The preliminary investigation of Ag^0^-aerogel granules that were aged in flowing dry air for up to six months and then loaded with iodine did not reveal any impact of aging on the aerogel microstructure or the silver nanoparticles in the aerogel, including their spatial distribution and morphology.^28^ In addition, exposure of the Ag^0^-aerogel to iodine during long-term static tests under vacuum did not show any effect of iodine on aging process. The sorbent retained its sorption capacity. Since iodine did not seem to effect aging, and considering big differences in iodine loading capacity for samples after aging in different environments with biggest drop observed for samples aged in flowing dry air containing 1 vol% NO, six samples of Ag^0^-aerogel were studied: (1) unaged, (2) aged in dry air, (3) aged in humid air, (4) unaged and then loaded with iodine, (5) aged in NO for 1 month and then loaded with iodine, and (6) aged in NO for two months and then loaded with iodine. The main purpose of the study reported here was to investigate, with an array of experimental methods, the unaged and aged samples of Ag^0^-aerogel with and without iodine to elucidate possible reasons leading to a loss of iodine loading capacity for this sorbent. The selected samples were analyzed with Brunauer–Emmett–Teller (BET) nitrogen adsorption to track the changes in specific surface area, total pore volume, and average pore size, with X-ray photoelectron spectroscopy (XPS) to track the changes in surface chemistry, and with scanning electron microscopy (SEM) and transmission electron microscopy (TEM) to visualize macro- and microstructural changes and distribution of individual elements.

## Results and discussion

### Unaged silver-functionalized silica aerogel loaded with iodine

**Fig. 1:**
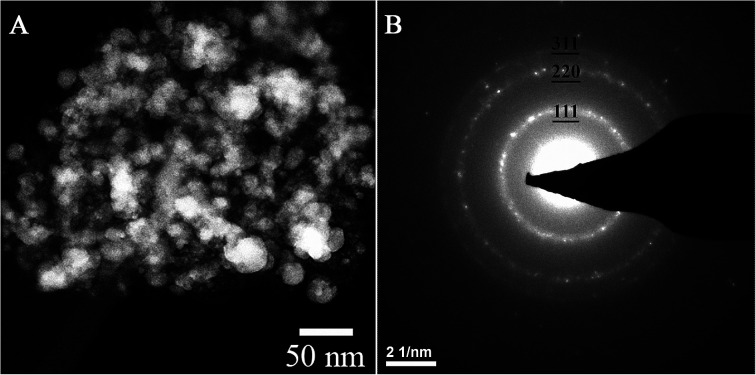
TEM image of AgI particles that were produced on Ag^0^-functionalized silica aerogel during an iodine sorption test (A) and their electron diffraction pattern (B).

Fig. 1 shows a TEM image of AgI nanoparticles for unaged iodine-loaded Ag^0^-functionalized silica aerogel and their representative diffraction pattern (Fig. 1B). Table 2 summarizes the quantitative analysis of the electron diffraction pattern for AgI nanoparticles. Comparison with structural characteristics of γ-AgI (right columns of Table 2) revealed that the *d*-spacing of AgI nanoparticles can be fully accounted for and indexed to a γ-AgI polymorph. The γ-AgI is a low-cubic modification that has been found to be stable below 147 °C. The other polymorphs of AgI, such as β-AgI (hexagonal phase, thermodynamically stable <147 °C) and α-AgI (high-cubic phase, thermodynamically stable >147 °C) were not present. The prevalence of γ-AgI over other modifications of AgI can be explained by similarities in the crystal structures of Ag and γ-AgI. In both, Ag is in face-centered cubic (FCC) arrangement. Therefore, the structural transformation of Ag to γ-AgI occurs readily due to lower activation energy than that for transformation to α-AgI or β-AgI. An attempt was made to investigate the possibility that some silver was not reacted with iodine and was present as silver metal. However, electron diffraction patterns could not discern this due to significant overlap of the silver planes with those of γ-AgI.

**Table 2:** Structural characteristics of AgI particle compared to γ-AgI (*F*4̄3*m*, *a* = 6.495 Å)^29^

Miller indices, *hkl*	Structural characteristics of AgI particle as determined from the ring pattern shown in Fig. 1		Structural characteristics of γ-Ag	
	Reciprocal diameter, nm^−1^	Lattice spacing, nm	Reciprocal diameter, nm^−1^	Lattice spacing, nm
111	5.3	0.377	5.3	0.375
220	8.6	0.232	8.7	0.230
311	9.9	0.198	10.2	0.196

### Effect of aging

Table 3 shows the BET results for four samples of Ag^0^-aerogel: (1) unaged and not loaded with iodine (sample A), (2) unaged and loaded with iodine (sample A-1), (3) aged in NO for one month and then loaded with iodine (sample B), and (4) aged in NO for two months and then loaded with iodine (sample C). The unaged Ag^0^-aerogel had a surface area of 84 m^2^ g^−1^, pore volume of 0.28 × 10^−3^ m^3^ kg^−1^, and adsorption/desorption (ads/des) pore size of 18/11 nm, respectively. After loading with iodine the unaged Ag^0^-aerogel exhibited surface area of 97 m^2^ g^−1^, the pore volume of 0.24 × 10^−3^ m^3^ kg^−1^, and the adsorption/desorption (ads/des) pore size of 15/10 nm, respectively. This small variation in surface area and pore volume can be explained by an error of measurement and by small degree of heterogeneity in the samples. However, noteworthy decrease in adsorption pore size can be attributed to captured iodine. The similar decrease was also observed for sample which was aged in NO for one month and then loaded with iodine. The sample had the surface area of 72 m^2^ g^−1^, the pore volume of 0.22 × 10^−3^ m^3^ kg^−1^, and ads/des pore size 15/10 nm. However, an additional decrease in ads/des pore size observed for a sample aged in NO for two months indicates that prolonged exposure to NO resulted in a decrease of pore size, which may lead to a decrease in the sorption capacity.

**Table 3:** Surface area, total pore volume, and average pore size for adsorption and desorption for unaged and NO-aged samples of Ag^0^-aerogel: (A) unaged and not loaded with iodine, (A-1) unaged and loaded with iodine (B) aged in NO for one month and loaded with iodine, (C) aged in NO for two months and loaded with iodine

Sample ID	Surface area, m^2^ g^−1^	Total pore volume, 10^−3^ m^3^ kg^−1^	Average pore size (ads/des), nm
A	84	0.28	18/11
A-1	97	0.24	15/10
B	72	0.22	15/10
C	78	0.21	10/6

**Fig. 2:**
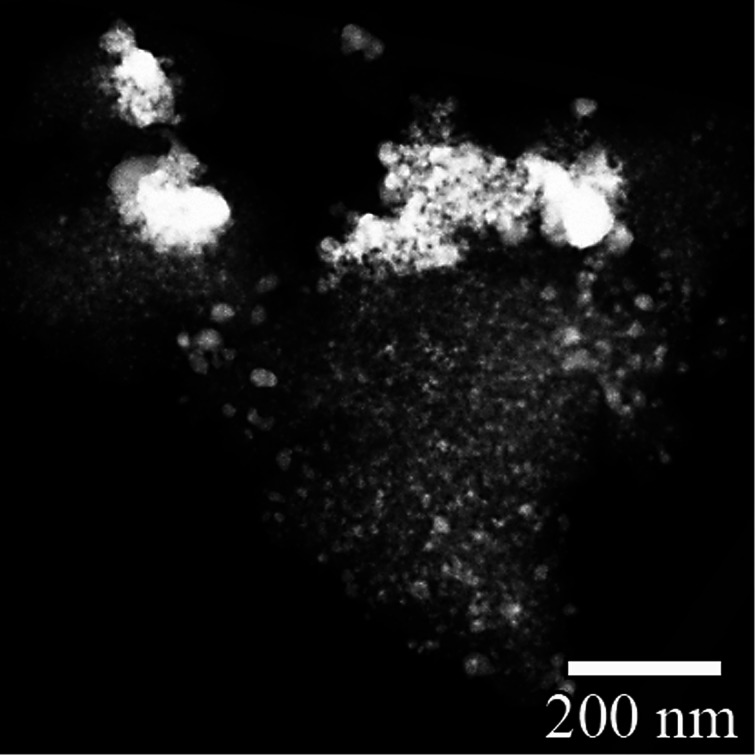
TEM image of Ag^0^-aerogel after aging for two months in NO, followed by loading with iodine; AgI nanoparticles and nano-sized inclusions are white and light gray, silica aerogel backbone is dark gray.

**Fig. 3:**
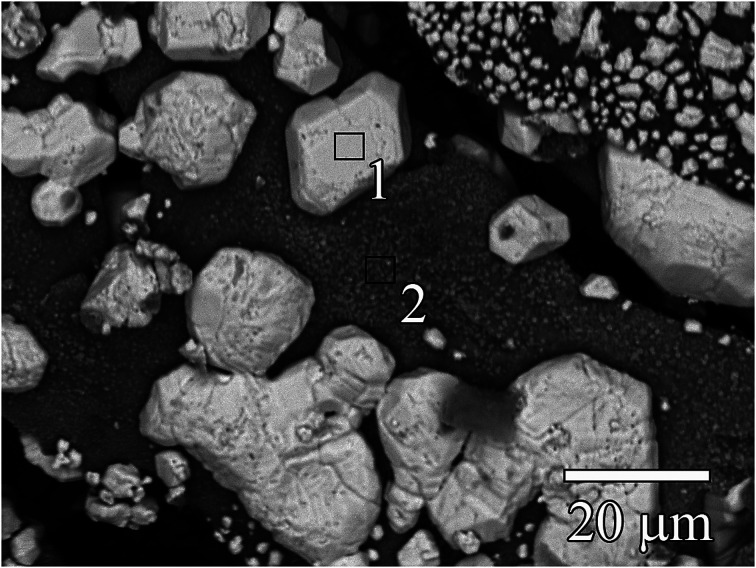
SEM image of Ag^0^-aerogel after one month of aging in NO followed by iodine capture showing AgI particles on silica aerogel backbone; AgI nanoparticles and micrometer-sized inclusions are light gray, silica aerogel backbone is dark gray. EDS in mass% for area 1: O – 29.41, Si – 6.07, S – 1.40, Ag – 36.41, and I – 26.70, and area 2: O – 44.38, Si – 34.17, S – 3.65, Ag – 10.71, and I – 7.09.

Fig. 2 shows a TEM image of Ag^0^-aerogel after two months of aging in NO followed by loading with iodine. While the nanoparticles of AgI in unaged iodine-loaded Ag^0^-aerogel were uniformly distributed on the silica aerogel backbone surface (Fig. 1), some agglomeration of AgI nanoparticles was observed in the NO-aged sample. The agglomeration of particles was also observed for Ag^0^-aerogel sample that was NO-aged for one month. Fig. 3 shows details for a couple of agglomerated AgI nanoparticles, including energy dispersive spectroscopy (EDS) analysis of the areas of the Ag^0^-aerogel aged for one month in NO. The particle agglomeration was only sporadic and the size of agglomerates varied from nano to micron-size inclusions for both samples. However, by considering silver loading of 246 mg per g of Ag^0^-aerogel, the average radius of silver nanoparticles of 5 nm, and the density of silver of 10.5 g cm^−3^, the total number of silver nanoparticle spheres per g of Ag^0^-aerogel, 4.49 × 10^16^, can be calculated. From analyses with TEM and SEM it can be estimated that there were approximately 10 000 agglomerates per g of Ag^0^-aerogel. If, for simplicity, each agglomerate contained about 10 000 nanoparticles, agglomeration accounted for 2.23 × 10^−7^% of the total number of silver nanoparticles. This would suggest negligible influence of agglomeration, induced by NO aging, on iodine capture performance. However SEM and TEM analysis also indicated that the size of individual silver nanoparticles increased. This would decrease their effective specific surface area and contribute to the loss of iodine loading capacity after NO aging.

### Effect of aging investigated with XPS

Determining Ag speciation with XPS can be problematic because Ag-sulfides, -oxides, and metallic species often have overlapping binding energies (BEs). However, here, we looked for consistency with known Ag BEs and coupled them to the mass balance of the corresponding S and I species. For example, three criteria were considered for identifying Ag and I in AgI: (1) the Ag and I have BEs consistent with AgI, (2) the atom concentration ratio of Ag(AgI) and I(AgI) is close to one, and (3) no other plausible species exist, as evidenced by a BE or mass balance mismatch. Since comparing absolute BEs to better than ±0.2 eV can be difficult for insulating materials, higher preference is placed on criteria 2 and 3. In addition, given the surface sensitivity of XPS, elemental ratios are often underestimated in larger particles relative to smaller particles and adsorbed species. Therefore, we sought additional support for the XPS results from TEM and SEM.

Table 4 summarizes species resolved by fitting XPS spectra as well as through their BEs and proportions. The Ag^0^ refers to metallic silver that occurs as nano-particles, where the BE is dependent on particle size. The terms Ag–S–r and r–S–Ag refer to the same species; the former represents the S component and the latter the Ag component. These species indicate the interaction of thiol groups with Ag atoms at the surface of the Ag^0^ particles. Thiol is the S in the (r)-HS functional groups that cap the organic chains (r) tethered to the SiO_2_ surfaces; S^+^ represents any S that is more oxidized than thiol but less oxidized than sulfate. The sulfate, SO_4_^2−^ (S^6+^), forms by oxidation of thiol groups; some sulfate remains in place and some is converted to Ag_2_SO_4_ (observed in samples aged in humid and dry air). The sulfate (1) and sulfate (2) have different BEs, but were not assigned to specific bonding environments. The AgI is the particulate solid phase, I-organic is tentatively assigned to I physisorbed to side chains of r, and I_*x*_O_*y*_ represents oxidized I of unknown physical form. Element atomic concentrations normalized to Si concentrations are listed in Table 5. Atom concentrations of the elements are normalized to Si in order to minimize variations in signal intensity due to potential differences in C over-layer thickness, sample preparation, and instrument performance. Elemental sensitivity factors were specifically tuned to the XPS instrument and frequently checked against standards.

**Table 4:** Binding energies and proportion of species for I, S, and Ag in unaged and aged samples with and without iodine

Sample	I3d			S2p			Ag3d		
	I species	I3d_5/2_ (eV)	%	S species	S2p_3/2_ (eV)	%	Ag species	Ag3d_5/2_ (eV)	%
Unaged				r–S–Ag	162.4	62.9	Ag–S–r	368.3	47.3
				Thiol	163.6	30.9	Ag^0^ < 2 nm	369.1	52.7
				Sulfate	169.0	6.2			
Unaged I loaded	AgI	619.9	63.9	Thiol	163.7	63.9	AgI	368.6	55.8
	I-organic	620.4	31.4	S^+^	166.2	8.6	Ag^0^ < 2 nm	369.1	44.2
	I_*x*_O_*y*_	621.4	3.4	Sulfate (1)	168.9	25.7			
				Sulfate (2)	170.0	2.3			
NO-aged, 1 month I loaded	AgI	619.7	82.6	Sulfate	169.3	100.0	AgI	368.2	61.2
	I-organic	620.2	15.6				Ag^0^ < 6 nm	368.7	39.8
	I_*x*_O_*y*_	621.1	1.8						
NO-aged, 2 months I loaded	AgI	619.8	88.6	Sulfate (1)	168.8	61.9	AgI	368.2	50.9
	I-organic	620.4	9.3	Sulfate (2)	170.0	38.1	Ag^0^ < 6 nm	368.7	49.1
	I_*x*_O_*y*_	621.9	2.1						
Humid air, 1 month				r–S–Ag	161.4	31.1	Ag_2_SO_4_	367.8	57.8
				Thiol	163.4	24.4	Ag–S–r	368.5	32.5
				S^+^	165.1	3.7	Ag^0^ < 2 nm	369.2	9.7
				Sulfate (1)	167.9	16.4			
				Sulfate (2)	169.2	24.4			
Dry air, 6 months				r–S–Ag	161.8	46.2	Ag_2_SO_4_ + Ag–S–r	368.3	53.6
				Thiol-like	163.2	11.4	Ag^0^ < 6 nm	368.8	34.2
				S^+^	164.6	5.8	Ag^0^ < 2 nm	369.5	11.5
				Sulfate (1)	168.0	19.7			
				Sulfate (2)	169.1	16.8			

**Table 5:** Atom concentrations normalized to Si

Sample	S/Si		I/Si		Ag/Si	
Unaged	Total	0.20			Total	0.27
	r–Ag–S	0.13			Ag–S–r	0.13
	Thiol	0.07			Ag^0^ < 2 nm	0.14
Unaged I loaded	Total	23.8	Total	0.31	Total	0.35
	Thiol	15.2	AgI	0.21	AgI	0.19
	S^+^	2.0	I-organic	0.09	Ag^0^ < 2 nm	0.15
	Sulfate (1)	6.1	I_*x*_O_*y*_	0.01		
	Sulfate (2)	0.5				
NO-aged, 1 month I loaded	Total	0.21	Total	0.158	Total	0.22
	Sulfate	0.21	AgI	0.131	AgI	0.13
			I-organic	0.025	Ag^0^ < 6 nm	0.08
			I_*x*_O_*y*_	0.003		
NO-aged, 2 months l loaded	Total	0.22	Total	0.070	Total	0.14
	Sulfate (1)	0.14	AgI	0.062	AgI	0.07
	Sulfate (2)	0.08	I-organic	0.007	Ag^0^ < 6 nm	0.07
			I_*x*_O_*y*_	0.001		
Humid air, 1 month	Total	0.21			Total	0.22
	r–S–Ag	0.07			Ag–S–r	0.07
	Thiol	0.05			Ag–SO_4_	0.13
	S^+^	0.01			Ag^0^ < 2 nm	0.02
	Sulfate (1)	0.04				
	Sulfate (2)	0.05				
Dry air, 6 months	Total	0.25			Total	0.46
	r–S–Ag	0.12			Ag–SO_4_ + Ag–S–r	0.24
	Thiol-like	0.03			Ag^0^ < 6 nm	0.16
	S^+^	0.01			Ag^0^ < 2 nm	0.05
	Sulfate (1)	0.05				
	Sulfate (2)	0.04				

**Fig. 4:**
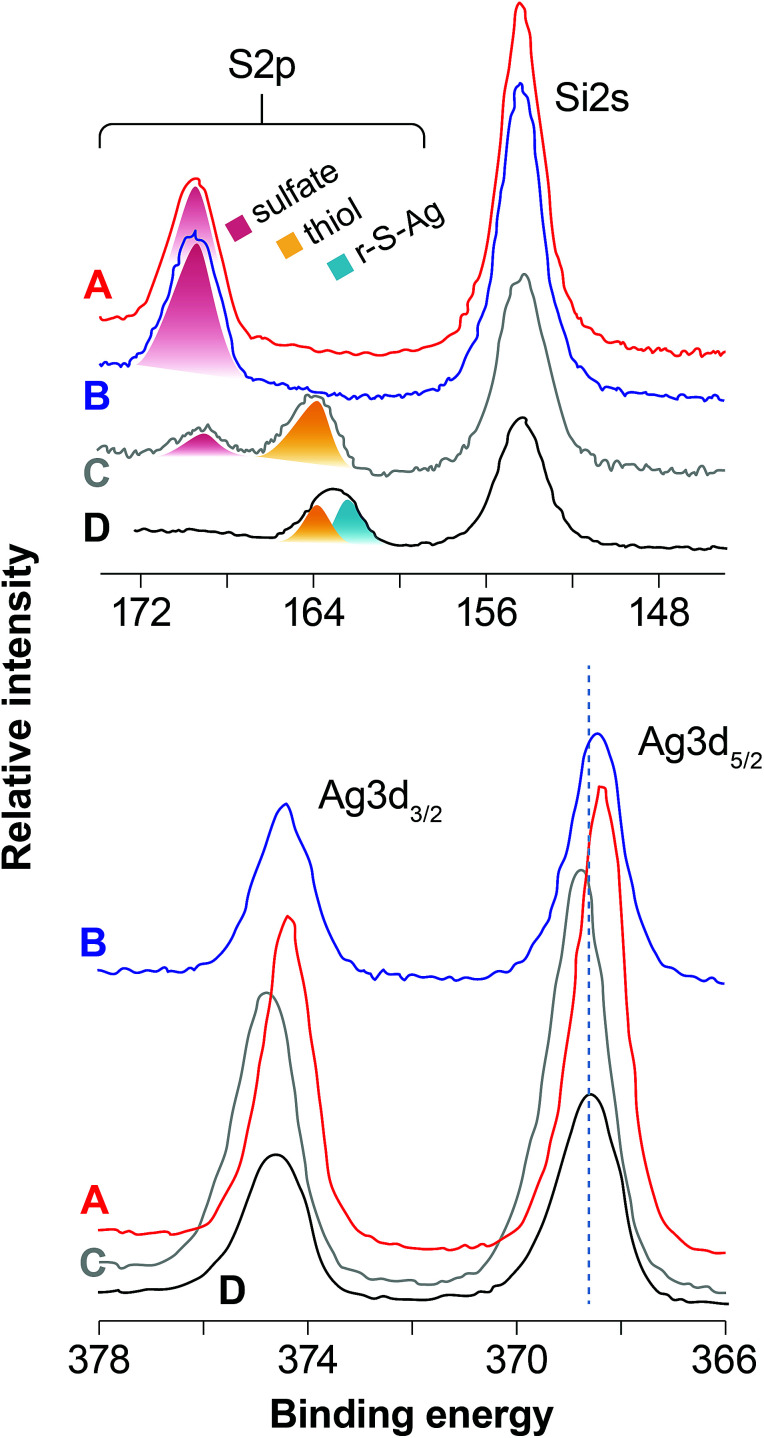
Regional scans of the S2p (top) and Ag3d (bottom) XPS spectra for aerogels that were (A) aged for 1 month in NO and loaded with iodine (B) aged for 2 months in NO and loaded with iodine, (C) unaged and loaded with iodine, and (D) unaged and not loaded with iodine. The labeled species sulfate, thiol, and r–S–Ag mark their approximate binding energies.

**Fig. 5:**
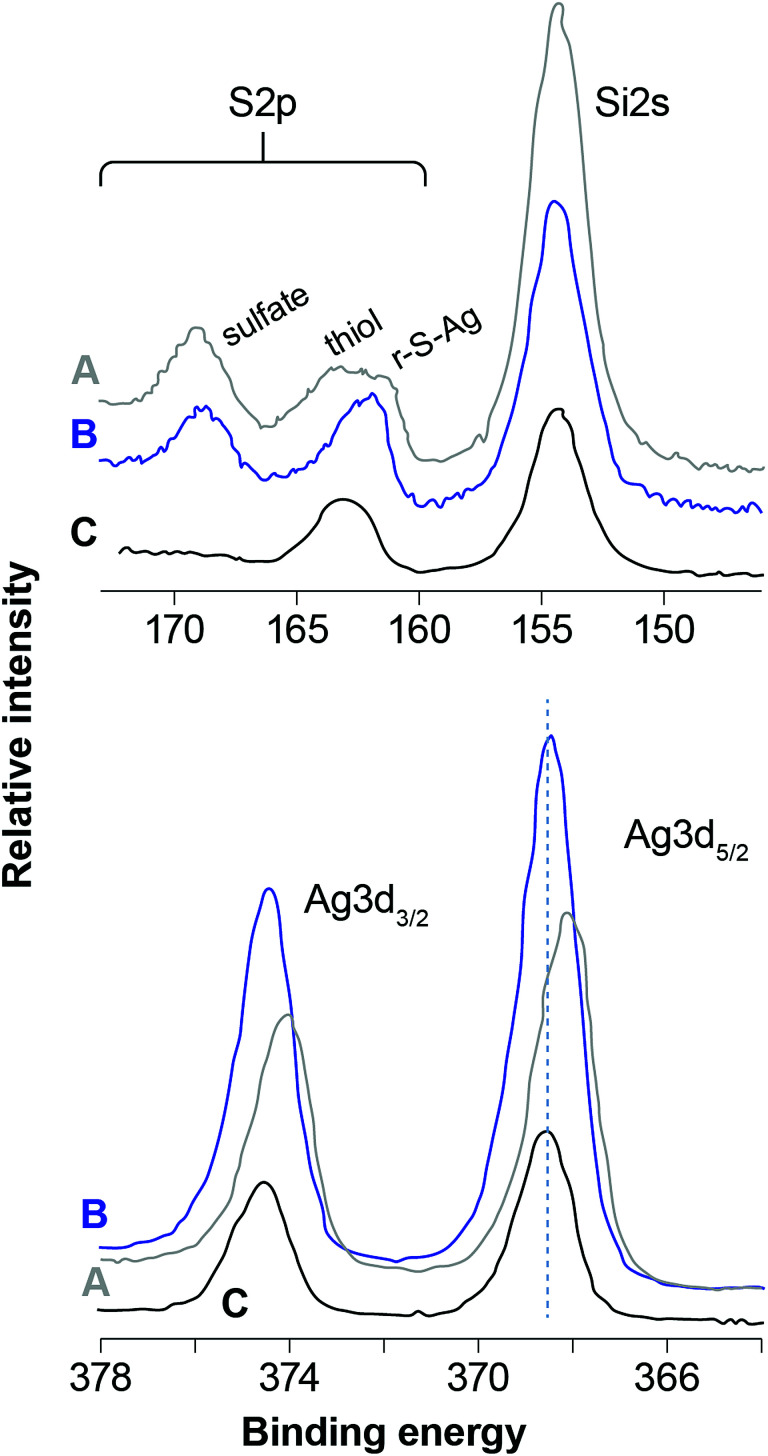
Regional scans of the S2p (top) and Ag3d (bottom) XPS spectra for samples not loaded with iodine: (A) 1 month humid air, (B) 6 months dry air, and (C) unaged. The highlighted peaks for sulfate, thiol, and r–S–Ag indicate their approximate locations and binding energies.

The BEs for Si2p (as well as Si2s) and O1s are consistent with a fully-polymerized SiO_2_ polymorph for each sample and the O/Si ratio is close to 2, which is the expected value for SiO_2_. Fig. 4 and Fig. 5 compare scans of the S2p–Si2s and Ag3d regions for the different samples. The I3d spectra were about the same except for intensity (mostly AgI, as discussed below) and are not plotted. Fig. 4 illustrates the effect of different aging conditions on samples loaded with I. Loading the unaged sample with I in dry air induces some S oxidation; contrastingly, S was quantitatively oxidized to a sulfate-like species in the NO-aged samples. More subtle differences are apparent for Ag3d, in which the BEs are lower for the NO-aged samples. The effect of humid- and dry-air aging for samples that were not loaded with I is shown in Fig. 5. Aging in humid or dry air splits the S2p peak at ∼164 eV (thiol in the unaged sample) into two peaks (a higher and a lower BE), and decreases the BE of Ag3d, marginally in dry air, but significantly for the sample aged in humid air.

**Fig. 6:**
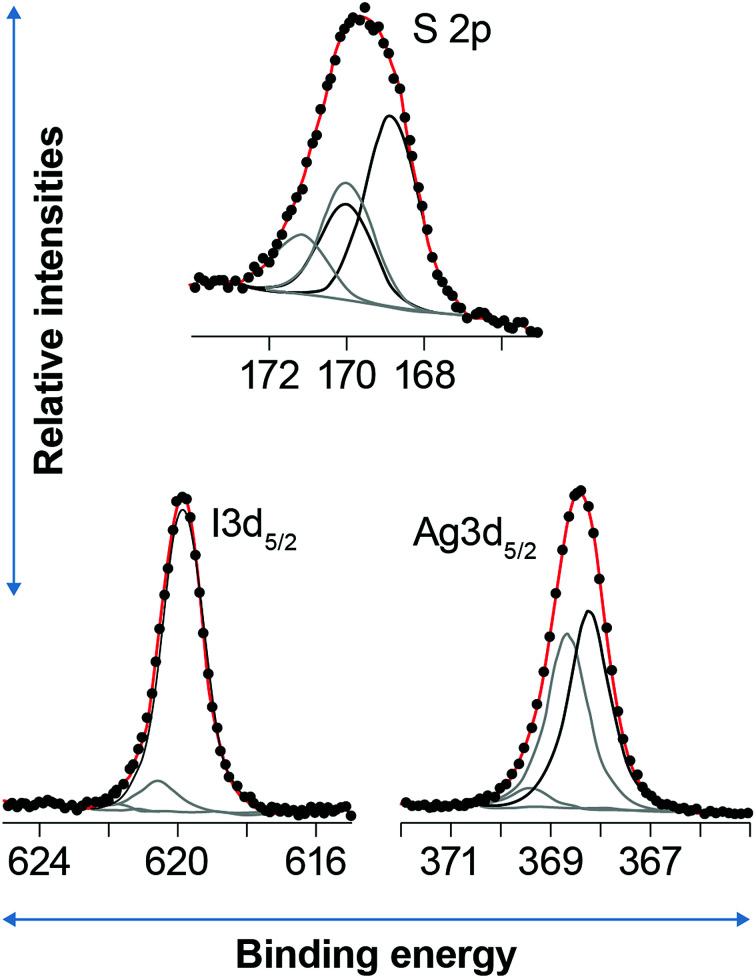
Example curve fits to the S2p (top), I3d_5/2_ (bottom left), and Ag3d_5/2_ (bottom right) XPS spectra for the aerogels aged in NO for 2 months followed by loading with iodine. The circles are data, the red curve is the fit envelope, and the black and gray curves are the different components as shown in Table 4.

Fig. 6 shows an example of a curve fitting the S2p, I3d_5/2_ and Ag3d_5/2_ peaks for the sample aged for two months in NO. Fitting S2p for the unaged sample that was not I-loaded yields components at 162.4 and 163.6 eV, with near-detection-limit sulfate at ∼169.0 eV. The components at 162.4 and 163.6 eV are assigned to r–S–Ag^30,31^ and free thiol^30,32^ species, respectively, consistent with the reducing conditions during synthesis of Ag^0^-aerogel. Fitting the Ag3d_5/2_ peak produced two major components at 368.3 and 369.1 eV. The component at 368.3 eV is assigned to Ag–S–r,^30,31^ consistent with the molar ratio of Ag(Ag–S–r)/S(r–S–Ag), nearly equal to 1 (Table 5). Given TEM evidence for Ag^0^ nanoparticles, it is plausible that the Ag–S–r species (where S is the thiol functional group and r is the organic chain tethered to SiO_2_) records Ag atoms at the surface of the Ag^0^ particles that interact with thiol groups. The component at 369.1 eV is consistent with an Ag-organic species^33,34^ and may represent Ag ions trapped in the organic matrix. Alternatively, given the strong dependence of Ag3d binding energy on the size of silver nano-particles, where smaller size yields higher Ag3d BEs,^35^ results suggest that there is a reservoir of Ag^0^ particles/clusters at <2 nm that could grade into individual Ag atoms/ions. Indeed, the Ag–S–r species might be associated with the interaction of the surfaces of these particles with S, as discussed above.

Loading the unaged sample with iodine oxidized an appreciable amount of thiol to sulfate (Fig. 5; Table 4). A majority of free thiol was preserved, with a minor amount of partially oxidized sulfur occurring at ∼166 eV; in contrast, the Ag–S–r species disappeared. Two sulfate species were resolved with the dominant one at 168.9 eV and a minor one at 170.0 eV; the presence of two species, rather one is not yet understood. The I3d peak was fit with three components at 619.9, 620.4, and 621.4 eV. The low BE component is close to the expected value for AgI and this assignment is confirmed given the following analysis: The Ag3d peak was best fit with two components at 368.6 and 369.1 eV. The high BE component is assigned to the same Ag^0^ < 2 nm fraction as the unaged, non-I loaded sample. Given that the ratio of the low BE components for Ag and I, Ag : I, is 0.93, within an error of 1, and that both have BEs near that for AgI, we assigned them to AgI (Table 2). The components at 620.4 and 621.4 eV are assigned to I-organic, and I_*x*_O_*y*_ species.^3,4^

**Fig. 7:**
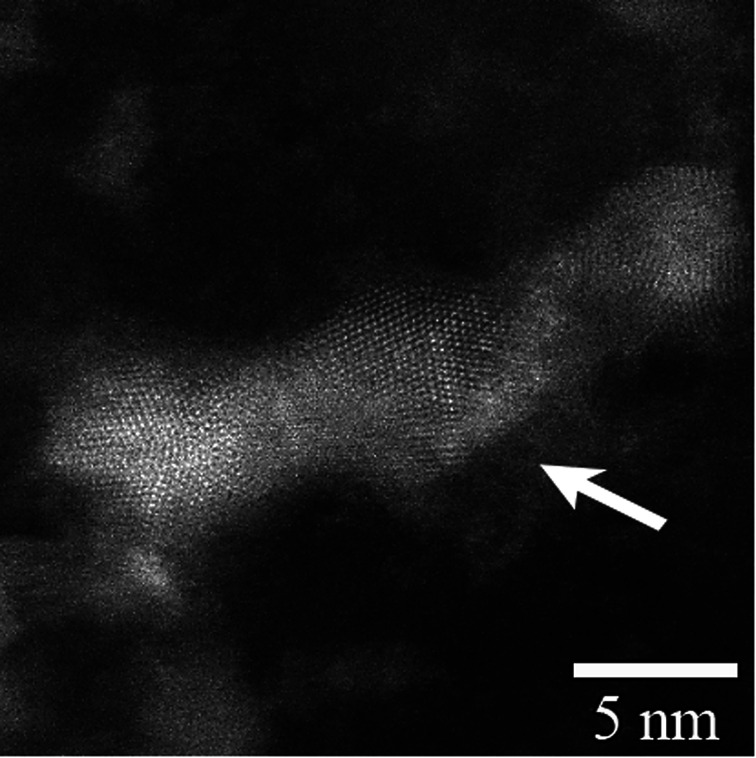
High-magnification TEM image of silver nanocluster found in Ag^0^-aerogel after aging in NO for 2 months followed by loading with iodine. The gray layer at the cluster boundary (indicated by arrow) contained a high concentration of sulfate.

The samples aged in NO for either 1 month or 2 months and then loaded with I record complete oxidation of S to sulfate. Fitting I3d and Ag3d yields similar species to those discussed for the unaged I loaded sample, albeit in somewhat different proportions (Table 4). The lower BEs for the Ag^0^ nanoparticles relative to those in the unaged I-loaded sample are interpreted to result from a size difference (Table 4), in accord with Salido *et al.*,^35^ and are partly responsible for the overall decrease in the Ag3d BE. There is no evidence for Ag_2_SO_4_ in any of the aged I-loaded samples, despite the presence of ample sulfate. The Ag is well accounted for by a combination of AgI and Ag^0^ nano-particulate phases (Table 5). It was hypothesized that sulfate forms by the oxidation of thiol groups (r–S) to yield r–SO_4_ functional groups. These sulfate groups likely interact with the Ag^0^ nano-particles to some degree and appear in patches surrounding Ag^0^ nano-particles (Fig. 7). These patches can partly or completely cover the surface of silver nanoparticles and act as a barrier, preventing a complete utilization of silver for iodine sorption. Two observations warrant further discussion: (1) I and Ag concentrations for the NO-aged samples are appreciably lower than those of the unaged I-loaded sample, in which aging at longer times in NO decreased concentrations even further (Table 5); and (2) all the I-loaded samples appear to contain appreciable amounts of Ag^0^ nano-particles and non-AgI I-species, indicating incomplete conversion to AgI. However, given that Ag is conserved, we suggest that XPS is likely overestimating the amount Ag^0^ particles and non-AgI I-species relative to AgI because it is a surface-sensitive spectroscopic technique. In other words, XPS is undercounting Ag and I in the bulk of the AgI particles relative to the smaller Ag^0^ nano-particles and adsorbed I species. Further, the nearly 50% decrease in total Ag and I after NO aging for 1 to 2 months is due mostly to a decrease in the AgI phase suggesting that Ag particle size increases with increased NO-aging times (Fig. 2 and 3).

Aging in humid and dry air (no I loading) yields a complex range of S oxidation states from sulfide (r–S–Ag) to sulfate (Table 4). Contrary to the NO-aged samples, there is also evidence for the formation of silver sulfate, which is the cause of the decrease in Ag BEs. The evidence is strongest for the sample aged in humid air, which is consistent with the presence of water assisting the diffusion of ions and subsequent nucleation and growth of silver sulfate.

Two XPS observations correlate with decreased efficiency of I capture by Ag after aging: (1) the increase in Ag^0^ particle sizes, and (2) the formation of sulfate, whether in the form of silver sulfate or oxidized thiol groups. Increasing Ag^0^ particle size during NO-aging would decrease reactive surface areas and lead to armoring, which could both slow the kinetics and reduce the extent of conversion to AgI. In cases where sulfate is present as oxidized thiol groups (*i.e.*, NO-aging), two mechanisms might be at work: (1) sulfate might stabilize Ag atoms at the surface of Ag^0^ particles, and/or (2) sulfate might hinder the diffusion of I_2_ to the surface of Ag^0^ particles.

In opposition to the above, Ag^0^ particle sizes do not appear to have increased during aging in humid air; however, there is evidence that some silver sulfate formed, which would lead to a decrease in the sorption capacity. Aging in dry air appears to increase Ag particle sizes to some degree (two populations were identified; Table 4) and possibly result in the formation of silver sulfate. However, the high Ag/Si ratio observed after aging in dry air suggests that Ag is well dispersed, which could bode well for restoring efficiency.

## Conclusions

It is important that the sorption performance of the sorbent for iodine does not change with time when the plant is idling and sorbent is exposed to flowing heated air containing water vapor and possibly NO_*x*_ gas. The Ag^0^-aerogel shows significantly higher resistance to corrosion gases compared to Ag^0^Z. However, exposure of this sorbent for extended time periods to flowing air containing 1 vol% NO at 150 °C results in oxidation of thiol to sulfate, accompanied by an increase of the size of individual silver nanoparticles and the spreading of sulfate on their surfaces. Both of these phenomena contribute to decreasing sorption performance in a gas stream containing NO. However, formation of silver sulfate appears to be the main factor in the decrease of sorption capacity for samples aged in dry or humid air.

A viable solution to overcome “aging” issue may be to expose the Ag^0^-aerogel to a reducing gas stream for a short period, which would reduce oxidized silver back to metal and sulfate to sulfide. However, sorption performance of Ag^0^-aerogel is not expected to be fully restored. Observed increase in the size of silver nanoparticles (decrease of their effective specific surface area) during aging process is not reversible and will likely continue during periods of aging, further decreasing iodine loading capacity of the sorbent. In addition, during the regeneration, when the sorbent is exposed to hydrogen at increased temperatures, silver nanoparticles are expected to growth as well.

## Materials and methods

### Ag^0^-functionalized silica aerogel

Silver-functionalized silica aerogel was synthesized using a previously developed procedure.^12^ Briefly, granules of silica aerogel from United Nuclear (Laingsburg, MI) were heat-treated at 400 °C for 60 min to remove trimethylsilyl groups (installed by manufacturer to make the aerogel hydrophobic) and hydrated in humidity-saturated air overnight. Following hydration, 3-(mercaptopropyl)trimethoxysilane [3-MPTMS; HS(CH_2_)_3_Si(OCH_3_)_3_, 95%] (Sigma Aldrich, St. Louis, MO) was distributed throughout the granules using a syringe at ∼30 mL per 17 g of unhydrated sample. The wetted material was loaded into a 1 L high-pressure vessel heated to 150 °C, the vessel was filled with supercritical CO_2_ at 24 MPa, and the sample cooked for 24 h. Following this process, the thiol-modified aerogel (∼12.5 g) was altered by installing the Ag(i) ions through treatment with 300 mL of 3.94 mass% AgNO_3_ (≥99%, Sigma Aldrich) solution. About 60 mL of methanol was added to the solution to facilitate wetting of the moderately hydrophobic thiol-modified aerogel. The silver nanoparticles were produced on the silica aerogel pore surfaces by reducing the silver thiolate adduct ions at 165 °C for 2 h under a 25 mL min^−1^ of 2.7% H_2_ in Ar in a glass column.

### Aging

Deep-bed test system was used to age the samples in flowing dry and humid air and in dry air containing 1 vol% NO. For each test, about 20 g of Ag^0^-aerogel was transferred into a 4.7 cm diameter stainless steel column located in the oven. The oven was heated to 150 °C and held at this temperature for the duration of the test. The gas velocity through the column was 3.5 m min^−1^. At selected time intervals the flow of gas was discontinued, the oven was turned off and the sample was allowed to cool to room temperature. Each time, the whole sample was removed from the column and gently mixed to ensure sample homogeneity. About 5 g were collected for analysis and the remaining material was returned back for continued aging.

### Iodine loading

A thermogravimetric analyzer was used to load the unaged and aged samples with iodine. It was configured from a modified VWR model 1330GM standard laboratory oven, a Mettler-Toledo Model XS603S balance (0.001 g accuracy), a three-legged weighing pan that extended out of the oven heat zone to the microbalance below, and a gas-mixing manifold with flow controllers and an iodine column. The three-legged weighing pan held the sample in a wire-bottomed basket. Surrounding the weighing pan was a glass cylinder with an open top. The mixture of gases from the gas mixing manifold entered the TGA oven and passed through a 20 ft long Teflon tube heat exchanger before entering the glass cylinder. The sample was first dried to a constant weight under 2 L min^−1^ N_2_ at 150 °C. Then, dry air was introduced and its flow gradually increased to 11.85 L min^−1^ (flow rate corresponds to a superficial velocity of 10.3 m min^−1^). After the samples weights were stabilized, 11.85 L min^−1^ air stream laden with 56 ppm of iodine was allowed to pass through the column and sample weight was recorded every minute for up to 24 h. The test was completed with a 24 h air purge to remove any physisorbed iodine. Only a negligible amount of physisorbed iodine was observed in tested samples.

### Specific surface area, pore volume, and pore size

Adsorption and desorption isotherms were collected with a Quantachrome Autosorb-6B (Quantachrome Instruments, Boynton Beach, FL) gas sorption system on samples degassed at 25 °C for 8 h while under vacuum. The samples were analyzed with nitrogen adsorption and desorption at 77 K. The surface area was determined from the isotherm with the five-point BET method.^36^ The Barrett–Joyner–Halenda (BJH) method^37^ was used to calculate the total pore volume and pore size distribution including average pore diameter.

### X-ray photoelectron spectroscopy

The measurements were performed with Physical Electronics Quantum 2000 Scanning ESCA Microprobe (Physical Electronics, Inc., Chanhassen, MN). The microprobe used a focused monochromatic Al Kα X-rays (1486.7 eV) source for excitation and a spherical section analyzer. The instrument has a 16-element multichannel detection system. A 105 W X-ray beam focused to 100 μm diameter was rastered over a 1.4 × 0.3 mm rectangle on the sample. The X-ray beam was incident normal to the sample and the X-ray photoelectron detector was at 45° off-normal. The high energy resolution data was collected using a pass-energy of 46.95 eV with a step size of 0.125 eV. For the Ag3d_5/2_ line; these conditions produced a full-width at half-maximum (FWHM) of 0.98 eV. Binding energies were referenced to Si2p at 103.4 eV. The S2p lines were best fit with a sufficient number of independent spin–orbit split doublets where the S2p_1/2_ and S2p_3/2_ peaks were given the same FWHM and 70 : 30 Gaussian : Lorentzian peak shape. The S2p_1/2_ level was set at half that intensity and positioned 1.18 eV above the S2p_3/2_ peak. Other lines of interest were fit with component peaks of equal FWHM and line shape, for which the remaining degrees of freedom were the component peak heights and the binding energies. The granules/powders of samples were deposited onto double-sided 3M Scotch brand tape supported by 1 × 3 cm flat Si wafers, which were then loaded into the XPS vacuum chamber for analysis.

### Scanning electron microscopy and energy dispersive spectroscopy

Sample imaging was performed using a JSM-7001F field emission-gun SEM (JEOL USA, Inc. Peabody, MA) in a low-vacuum mode at an accelerating voltage of 15 kV to minimize beam penetration. The samples were adhered to double-stick carbon tape and analyzed for changes in the morphology of particles. A Bruker xFlash® 6|60 detector (Bruker AXS Inc., Madison, WI) using ESPRIT (v2.0) was used for elemental spot analysis to determine the distribution of individual elements in different areas of the samples.

### Transmission and scanning transmission electron microscopies and selected area diffraction

The microstructural investigation was performed with an aberration-corrected FEI Titan 80-300 electron microscope (FEI, Hillsboro, OR) in scanning transmission electron microscopy (STEM) mode. The probe convergence angle was set to approximately 18 mrad and the inner detection angle on the high-angle annular dark field detector was approximately three times higher than the probe convergence angle. Compositional analysis was performed with an EDAX Si (Li) EDS detector. The obtained spectra were analyzed with FEI TIA software. Samples for TEM observations were prepared by dispersing a dry powder on lacey-carbon coated 200 mesh Cu TEM grids, which were then loaded into the TEM vacuum chamber for analysis.

## Author contributions

The manuscript was written through contributions of all authors. All authors have given approval to the final version of the manuscript.

## Funding sources

This work was funded by the Department of Energy Office of Nuclear Energy.

## Conflicts of interest

There are no conflicts to declare.

## Supplementary Material
